# Sequential occurrence of basal cell carcinoma in symmetrically identical positions of both lower eyelids: A rare finding of a common skin cancer

**DOI:** 10.4103/0974-620X.71895

**Published:** 2010

**Authors:** Shanti Pandey, Vimlesh Sharma, G. S. Titiyal, Vivekanand Satyawali

**Affiliations:** Department of Ophthalmology, Uttarakhand Forest Hospital Trust and Medical College, Kumaun University, Nainital -263 139, Uttarakhand, India; 1 Department of Medicine, Uttarakhand Forest Hospital Trust and Medical College, Kumaun University, Nainital -263 139, Uttarakhand, India

**Keywords:** Basal cell carcinoma, bilateral symmetrical position, eyelids

## Abstract

Basal cell carcinoma (BCC) is the most common type of skin cancer in white-skinned individuals but is rare in blacks and Indians. There are only few case reports about bilateral BCC of lower eyelids. Here we present a case of BCC appearing sequentially in symmetrically identical positions in both lower eyelids. The patient was a resident of high altitude and had worked out doors for seven to eight hours every day. Environmental and occupational parameters may have an important role to play in this context. There was no evidence of local invasion or distant metastasis.

## Introduction

Basal cell carcinoma (BCC) is the most common type of skin cancer common in white-skinned individuals but is rare in blacks and Indians. Skin cancers mainly affect sun-exposed areas like the neck and face (88–90%).[1] BCC rarely metastasizes or kills, but is still considered as malignant because it can cause significant destruction and disfigurement by invading surrounding tissues. Here, we present a rare case of BCC appearing sequentially in symmetrical positions in both lower eyelids, in a female resident of the hilly region of Kumaun (Uttarakhand), India. There are only few case reports of bilateral BCC of lower eyelids.[2,3]

## Case Report

A 51-year-old female, field worker, fair in complexion, and resident of the hilly region of Kumaun, India reported in March 2009 with a gradually enlarging mass in lateral portion of the right lower lid for last five years. The mass had rapidly increased in size in the three months prior to presentation. The patient also gave a history of itching, and occasional bleeding from the mass. There was a past ocular history of a similar mass in the left lower lid in an identical position, which appeared when she was around 20 years of age. It was excised at the age of 25 years for cosmetic reasons when it was pea-sized. Histological examination was not done at that time. After excision, the mass again recurred at the same location, continued to increase in size, and was associated with itching and occasional bleeding. Excision of mass with cheek rotation flap was done in 2001. Histological examination of this tissue revealed pigmented nodular variety of BCC of left lower lid.

The patient had been diagnosed with hypertension and diabetes three years back and was on irregular treatment. Family history of skin carcinoma was negative.

**Figure 1:**
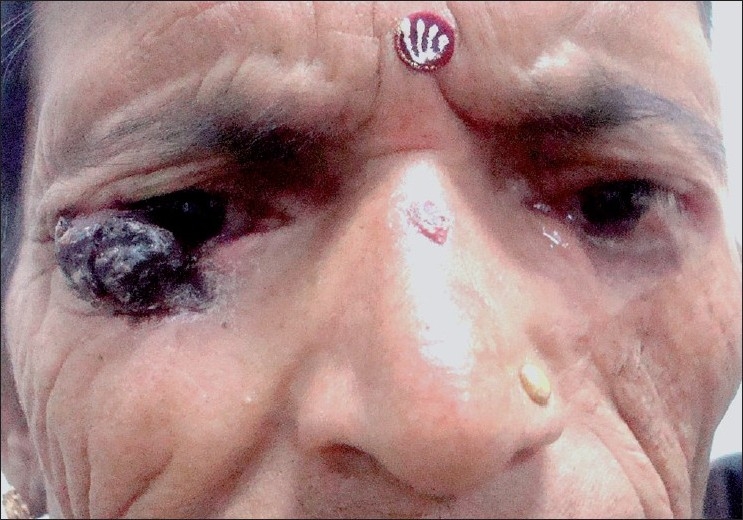
Nodular ulcerative mass in lateral half of right lower lid. Note scar mark after excision of a similar mass in a symmetrically identical position in the left lower lid.

Examination of the right lower eyelid revealed an approximately 25 × 30 mm, pigmented ulcerated, and nodular mass in the lateral half. It was painless, firmly adherent to the lid, and restricting the active movements of lower lid. The lower lid was slightly everted due to weight of this mass [Figure 1]. Clinically there was no evidence of regional lymphadenopathy. Examination of the left eye revealed loss of most of the lower lid with scar tissue in the infraorbital and temporal part of the left cheek. Some redness was noticed in conjunctiva adjacent to this region due to incomplete lid closure [Figure 1].

Systemic examination was unremarkable. Routine investigation revealed raised fasting and postprandial blood sugar levels, a normal hemogram, liver and kidney function tests. Chest X-ray and ultrasonography of abdomen did not reveal any abnormality.

### Histology

**Figure 2:**
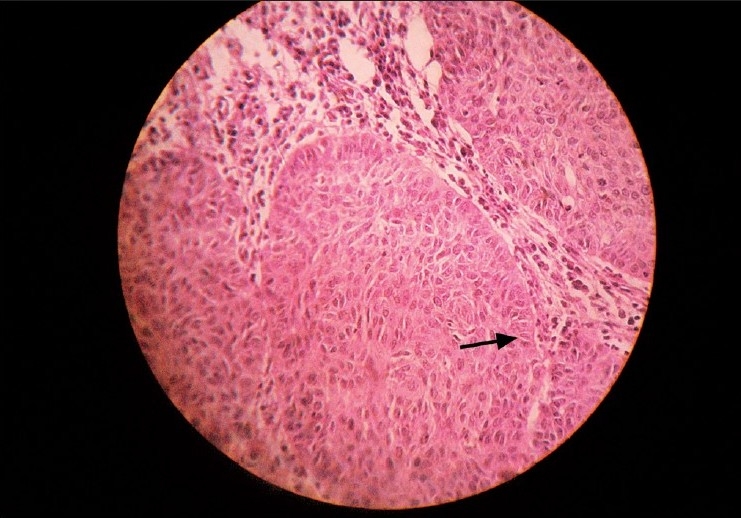
Histological picture (40x, oil immersion, hematoxylin and eosin staining) - Solid nests of darkly staining basal cells of the epidermis extending into the dermis with intensely dark blue stain of nuclei with hematoxylin. Typical palisade arrangement of cells at periphery of nests (arrow)

An incisional biopsy was performed prior to definitive excision to confirm the clinical diagnosis of a periocular malignant mass. Histological examination of the tumor in the right eyelid revealed solid nests of darkly staining cells extending into the dermis. All the cells were similar to the basal cells (basaloid) of the epidermis and few nuclei showed abnormal mitotic figures, which took up an in-tensely dark blue stain with hematoxylin. The cells at the periphery of the solid masses showed a typical palisade arrangement [Figure 2]. These features led to the diagnosis of BCC of right lower lid.

### Treatment

**Figure 3:**
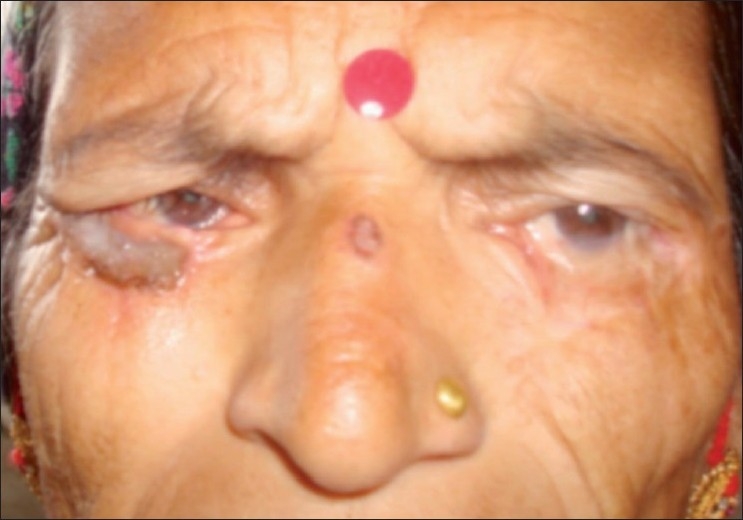
Photograph four weeks after excision of the lid mass and right eyelid reconstruction

Clinically, a rough estimate of tumor margins were made with the skin of the right lower eyelid on stretch using the following guides - transition in surface contour; altered vascularization (fewer capillaries and more telangectasia); altered skin color; tumor depth was judged by the lesion’s mobility over underlying tissues. Standard surgical excision with frozen section control was done. A two-staged lower eyelid reconstruction was done with tarsoconjunctival flap and full thickness skin graft taken from the thigh [Figure 3]. The tumor was excised with 2 mm margins, and repair delayed for two days, providing time for histological confirmation of complete excision with formal paraffin sections.[4]

## Discussion

BCC is the most common malignant eyelid tumor in the Caucasian population. The lower eyelid and medial canthus are the most frequent sites of origin.[5] About 95% of BCC occur between the age of 40–79 years with an average age at diagnosis of 60 years.[6] Cases of BCC in younger and older patients have been reported (27 months to 90 years).[7,8] In our case, the lesion appeared at the age of 20 years and gradually increased in size for 30 years, when it was excised and diagnosed as a nodular BCC. BCC is a slow growing, locally invasive and destructive tumor, and rarely metastasizes (0.0028–0.5%); thus, a metastatic workup often is not indicated.[9] But rare cases with metastatic potential have been described in literature.[10]

Forty percent of patients with BCC have an increased risk of developing second skin cancers, in five years.[11] In our patient, a second lesion appeared three years after the removal of first lesion. But the unusual feature in our case was appearance of second lesion in the identically symmetrical position in the right lower lid. There are few case reports of bilateral BCC of lower eyelids.

Hyde (1906) recognized UV radiation as a carcinogen, which is currently believed to be the most important cutaneous carcinogen with both initiating and promoting effects especially at 290–320 nm wavelength.[12] Potential increase in environmental UV radiation at high altitude due to depleted ozone layer in space may increase the incidence of skin cancer. Our patient was a resident of high altitude and a field worker who used to work out door for long hours every day. BCC is more likely to occur in fair-skinned individuals[13] and the length of actinic exposure plays a direct role in the frequency of tumor occurrence. Genetic (e.g. Xeroderma pigmentosa) and ionizing radiation has also been implicated as a risk factor for development of BCC. Environmental and occupational parameters along with pigmentary factors of skin might have played an important role in appearance of BCC in our patient.

Clinically, BCC can be grouped into three types: nodular, nodulo-ulcerative (Rodent ulcer) and morpheaform (sclerosing type). Nodular BCC is the most common variety, appears as pink or pearly papule with overlying telangactesias. Black or brown pigmentation may also present. In our patient, it was black-pigmented nodular type of BCC. Nodulo-ulcerative type results from central ulceration of nodule. It is surrounded by rolled up borders, often described as Rodent ulcer. Morpheaform type of BCC is least common and appears as flat, indurated, yellow- pink plaque with ill-defined borders. The nodular subtype of BCC should be regarded as a potentially invasive and recurrent tumor. Histopathological examination and subtyping of all BCC tumors is recommended.[14] Differential diagnosis of BCC are other malignant lesions, adenexal tumors and cysts, and inflammatory/ infectious condition. Surgical treatment is generally accepted as the treatment of choice for removal of BCC.

## Conclusion

We have reported a rare occurrence of BCC in a bilaterally symmetrical position of the lower eyelids in a resident of hilly region of Kumaun, India. The initial appearance at a younger age is another unusual feature in our patient.
